# Supplementary material for: Longitudinal trends and determinants of patient-reported side effects on ART–a Swedish national registry study
Source: PLoS One. 2020 Dec 23;15(12):e0242710. doi: 10.1371/journal.pone.0242710 (PMC7757802; doi:10.1371/journal.pone.0242710)
Supplement: S1 Appendix — (DOCX) [file pone.0242710.s001.docx]

**S1 Appendix.**

**The Health Questionnaire in English**

*Please tick the appropriate box for each statement*

| **1. How satisfied are you with your physical health?** | | | | | |
| --- | --- | --- | --- | --- | --- |
| Very unsatisfied | Unsatisfied | Rather unsatisfied | Rather satisfied | Satisfied | Very satisfied |
| □ | □ | □ | □ | □ | □ |
| **2. How satisfied are you with your psychological wellbeing?** | | | | | |
| Very unsatisfied | Unsatisfied | Rather unsatisfied | Rather satisfied | Satisfied | Very satisfied |
| □ | □ | □ | □ | □ | □ |
| **3. How satisfied are you with your sexual life (regardless if you have sex with a partner or on your own)?** | | | | | |
| Very unsatisfied | Unsatisfied | Rather unsatisfied | Rather satisfied | Satisfied | Very satisfied |
| □ | □ | □ | □ | □ | □ |

**4a. Are you currently taking HIV medication?**

Yes □ Go to question 4b. No □ Go to question 5.

**4b. Do you experience any side effects?**

Yes □ Go to question 4c. No □ Go to question 4d.

| **4c. To what extent are you troubled by medical side effects?** | | | | | |
| --- | --- | --- | --- | --- | --- |
| Very troubled | Troubled | Rather troubled | Not very troubled | Not at all troubled |  |
| □ | □ | □ | □ | □ |  |

**4d. How many doses have you missed the last week?**

□ 0 □1-2 □ 3 or more doses

| **5: Do you feel involved in the planning and realization of your HIV care and treatment?** | | | | | |
| --- | --- | --- | --- | --- | --- |
| Never | Seldom | Sometimes | Always |  |  |
| □ | □ | □ | □ |  |  |

| **6. How satisfied are you with the quality of care provided at your HIV clinic?** | | | | | |
| --- | --- | --- | --- | --- | --- |
| Very unsatisfied | Unsatisfied | Rather unsatisfied | Rather satisfied | Satisfied | Very satisfied |
| □ | □ | □ | □ | □ | □ |

**Thank you for your participation!**
