# Supplementary material for: Longitudinal trends and determinants of patient-reported side effects on ART–a Swedish national registry study
Source: PLoS One. 2020 Dec 23;15(12):e0242710. doi: 10.1371/journal.pone.0242710 (PMC7757802; doi:10.1371/journal.pone.0242710)
Supplement: S1 Interview guide — (DOCX) [file pone.0242710.s002.docx]

S1 Interview guide – side effects hiv medication

**Swedish**

Vad tänker du på när du hör ordet biverkningar?

*Om informant räknar upp symptom*: vad tänker du att det beror på?

Har du biverkningar?

Vad tänker du är en biverkan?

Om du skulle förklara vad ordet biverkningar betyder, hur skulle du göra det?

Hur mycket påverkar biverkningar dig i din vardag?

**English**

What do you think about when you hear the word side effects?

Do you have side effects from hiv medication?

*If participant list symptoms:* What do you think caused those symptoms?

How would you explain the word side effects?

How much does side effects interfere with your everyday life
